# Effects of Liposome Composition and Size on Protein Corona Formation: Competitive Replacement of Abundant Proteins by High‐Affinity Proteins on the Liposome Surface

**DOI:** 10.1002/adhm.202505685

**Published:** 2026-01-15

**Authors:** Hwankyu Lee

**Affiliations:** ^1^ Department of Chemical Engineering Dankook University Yongin‐si South Korea

**Keywords:** binding free energy, cancer drug delivery, diffusivity, liposome‐protein interaction, molecular dynamics simulation, protein corona, vroman effect

## Abstract

Plasma proteins (human serum albumin, immunoglobulin gamma‐1, complement C3, and fibrinogen) are simulated with 26 and 36 nm‐sized cationic, anionic, and zwitterionic liposomes in water using coarse‐grained models. Proteins, initially placed randomly around a liposome, adsorb more significantly onto charged liposomes than onto zwitterionic liposomes through electrostatic interactions with lipid headgroups and hydrophobic interactions with lipid tails, with the greatest adsorption observed on cationic liposomes, in agreement with experimental observations. For zwitterionic liposomes, adsorption is more pronounced on liposomes with smaller headgroups, which also agrees well with experiments. Fewer serum albumin proteins adsorb onto smaller liposomes, consistent with experimental observations, because their relatively weak binding makes them more easily detached at higher bilayer curvature. In simulations of 32 proteins from four different species, although cationic and anionic proteins adsorb onto both cationic and anionic liposomes, they preferentially adsorb to oppositely charged liposomes, indicating the influence of both liposome and protein electrostatics. In particular, diffusivity and binding free‐energy calculations show that liposome‐protein interactions are energetically stabilized by the replacement of abundant proteins by high‐affinity proteins via electrostatic and hydrophobic protein‐protein and protein‐liposome interactions, to an extent dependent on protein type, supporting the Vroman effect of competitive protein adsorption.

## Graphical abstract

Coarse‐grained MD simulations capture the effects of liposome composition and size on protein adsorption onto the liposome surface, in agreement with experiments. Liposome‐protein interactions are energetically stabilized by the replacement of abundant proteins with high‐affinity proteins on the liposome surface through electrostatic and hydrophobic interactions, supporting the Vroman effect regarding competitive protein adsorption at the early stage of corona formation.

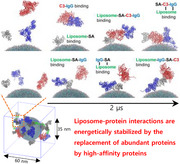

## 1 Introduction

Synthetic carriers such as dendrimers, hydrogels, nanosheets, and polymeric or metallic nanoparticles have been extensively investigated for the delivery of genes, drugs, and sensing molecules to target cells, although their cytotoxicity and immunogenicity often limit their applicability as drug delivery systems [1, 2, 3, 4, 5, 6]. In contrast, liposomes, which are artificially prepared vesicles composed of biocompatible phospholipids, have already achieved commercial success in gene and drug delivery [7, 8, 9, 10, 11]. For instance, liposome‐based vaccines enabled efficient delivery of messenger RNA during the coronavirus disease 2019 (COVID‐19) pandemic [12]. When administered into the bloodstream, liposomes are rapidly exposed to hundreds of plasma proteins [13], which adsorb onto their surfaces to form a so‐called “protein corona” [14, 15, 16]. The protein corona significantly alters the size and surface properties of liposomes, influencing their pharmacokinetics and cellular interactions [14, 15, 16]. To mitigate this effect, liposomes are frequently modified with hydrophilic polymers such as polyethylene glycol (PEG), a process known as PEGylation [17, 18]. While PEGylation reduces protein adsorption, excessive PEG decoration destabilizes the bilayer, restricting the amount of PEG that can be grafted and thereby failing to completely prevent protein binding [19, 20]. Importantly, the composition of the adsorbed proteins can be tuned by varying liposome components and surface properties, enabling the possibility of directing drug carriers toward specific cells [21]. Understanding how liposome composition and surface characteristics regulate protein adsorption is therefore crucial and has been the focus of numerous experimental and computational studies.

Experimental studies on the influence of liposome composition on protein adsorption were first pioneered by Caracciolo and colleagues [22, 23], showing that the composition of adsorbed proteins varies with plasma concentration under both in vitro and in vivo conditions [24], which affects cellular internalization pathways [25, 26]. They systematically characterized the types and amounts of plasma proteins bound to liposomes, showing substantially greater protein adsorption on charged liposomes than on neutral ones [27, 28, 29]. In particular, liposomes rich in zwitterionic lipids with small headgroups preferentially adsorb apolipoproteins and serum albumin, whereas those with high cholesterol content attract more immunoglobulins and complement proteins [29, 30]. Overall, protein adsorption follows the order cationic > anionic > neutral liposomes, with cationic liposomes exhibiting significantly higher protein binding [31]. Yang et al. further observed that immunoglobulins and complement proteins are among the most abundant species adsorbed onto neutral liposomes [32]. Mateos‐Maroto et al. showed that cellular uptake is controlled by adsorbed protein and liposome compositions [33]. Charbonneau and Tajmir–Riahi observed stronger binding of serum albumin to cationic lipids than to zwitterionic lipids through hydrophilic and hydrophobic interactions [34]. Similarly, Amarandi et al. recently found enhanced adsorption of serum albumin onto cationic liposomes compared with neutral ones, supported by their simulations of ion‐lipid bilayer interactions [35]. Regarding the effect of particle size, less protein adsorption has been observed on highly curved surfaces of gold particles [36, 37], although their surface properties differ from those of liposomes.

To complement experimental observations and interpret the mechanisms of protein corona formation at the atomic resolution, molecular dynamics (MD) simulations have been performed [38, 39, 40], showing the competitive adsorption and desorption of proteins (the Vroman effect) [41, 42, 43, 44, 45, 46, 47, 48, 49, 50, 51, 52, 53, 54], the influence of pH and ionic conditions on corona formation [55, 56, 57, 58], and how protein concentration and ionic strength modulate nanoparticle aggregation [59, 60, 61, 62, 63]. Most of these simulations have examined polymeric or metallic nanoparticles, whereas only a limited simulations have explored lipid‐based nanoparticles with protein corona. Li et al. characterized protein adsorption levels on polystyrene versus PEG‐coated nanoparticles [64], and Tang et al. found that apolipoproteins bind more strongly to silica nanoparticles in the presence of cholesterol than in its absence [65]. Niemela and Koivuniemi modeled the interaction of lecithin: cholesterol acyltransferase (LCAT) with lipid nanodiscs containing apolipoprotein‐mimetic peptides, rationalized by hydrogen bonding and salt‐bridge formation [66]. Our group observed that protein adsorption on PEGylated lipid bilayers decreases as PEG chains undergo the conformational change from the mushroom to the brush regime [67], and that proteins exhibit stronger interactions with anionic membrane leaflets than with zwitterionic leaflets [68]. Recently, our free‐energy calculations showed stronger adsorption of serum albumin to membranes with smaller headgroups and stronger adsorption of apolipoproteins to anionic membranes than to cationic or zwitterionic membranes, indicating a dependence on lipid headgroup size and charge [69]. Although these simulations have revealed the preferential adsorption of various plasma proteins onto specific lipid membranes, entire liposomes composed of different lipids in explicit water, particularly those larger than 30 nm that more realistically represent membrane curvature, have not yet been systematically simulated. In particular, competitive replacement of abundant proteins by high‐affinity proteins (the Vroman effect) has been simulated using implicit water and simple models [46, 70], but not at atomic resolution that can explicitly distinguish individual amino acids and liposome lipid headgroups.

In this work, we therefore report coarse‐grained (CG) MD simulations of liposomes mixed with multiple major plasma proteins such as human serum albumin (SA), immunoglobulin gamma‐1 (IgG), complement C3 (C3), and fibrinogen (FG). First, we equilibrate 26 and 36 nm‐sized liposomes in explicit water with 0.15 m NaCl, composed of cholesterol and phospholipids such as anionic 1,2‐dioleoyl‐sn‐glycero‐3‐phosphoglycerol (DOPG), cationic 1,2‐dioleoyl‐3‐trimethylammonium‐propane (DOTAP), zwitterionic 1,2‐dioleoyl‐sn‐glycero‐3‐phosphocholine (DOPC) or 1,2‐dioleoyl‐sn‐glycero‐3‐phosphoethanolamine (DOPE), which are commonly used in synthetic liposome experiments [71, 72, 73]. These equilibrated liposomes are then simulated with 4∼15 single protein species, showing protein adsorption onto the liposome surface, to an extent dependent on liposome composition and size, favorably compared with experimental observations. To more realistically represent competitive protein adsorption under human plasma conditions, 32 proteins consisting of four different species are simulated with each liposome, showing preferential adsorption of specific proteins onto cationic or anionic liposomes. In particular, diffusivity and binding free‐energy calculations show that liposome‐protein interactions are energetically stabilized by the replacement of abundant proteins with high‐affinity proteins on the liposome surface, which is rationalized by electrostatic and hydrophobic protein‐protein and protein‐liposome interactions. We will show that these findings help explain experimental observations regarding the effects of liposome composition and size on protein adsorption, as well as the Vroman effect.

## 2 Results and Discussion

### 2.1 Equilibration of 26 nm‐ and 36 nm‐sized Liposomes

**FIGURE 1:**
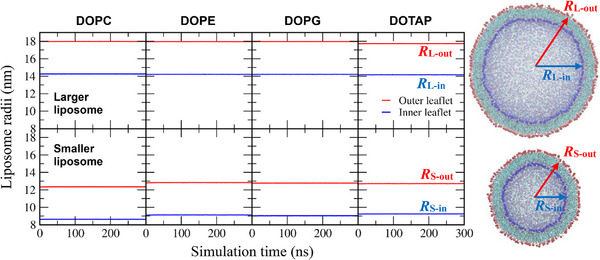
Inner and outer radii of liposomes, defined as the average distance between the liposome center and lipid‐phosphate beads (or trimethylammonium beads for DOTAP) in the inner and outer leaflets (respectively, blue and red colors), as a function of time. Final cross‐sectional snapshots of a larger liposome (*R*_L‐out_ = ∼18 nm, top) and a smaller liposome (*R*_S‐out_ = ∼13 nm, bottom) are shown. Phosphate beads of the inner and outer leaflets are highlighted as blue and red dots, respectively. Water and ions are omitted for clarity. Images were generated using Visual Molecular Dynamics [117] and MartiniGlass [118].

Small unilamellar vesicle (SUV) liposomes, composed of cholesterol and phospholipids such as DOPC (zwitterionic headgroup), DOPE (smaller zwitterionic headgroup), DOPG (anionic headgroup), or DOTAP (cationic headgroup), were equilibrated in explicit water containing 0.15 m NaCl for 300 ns. To investigate the effect of surface curvature, liposomes of two different sizes were simulated. The inner and outer radii of the liposomes, defined as the average distance from the liposome center to the lipid‐phosphate beads (or trimethylammonium beads for DOTAP) in the inner and outer leaflets, were calculated as a function of simulation time. In Figure 1, the radii remain stable throughout the simulations, indicating that the liposomes are well equilibrated within the simulated timescale. For the larger liposomes, the inner and outer radii are approximately 14 and 18 nm, respectively, while for the smaller liposomes they are about 9 and 13 nm, corresponding to a bilayer thickness of ∼4 nm. Hereafter, we refer to the smaller and larger liposomes as the 26 and 36 nm liposomes, respectively.

### 2.2 Adsorption of Single Protein Species Onto the Liposome Surface

**FIGURE 2:**
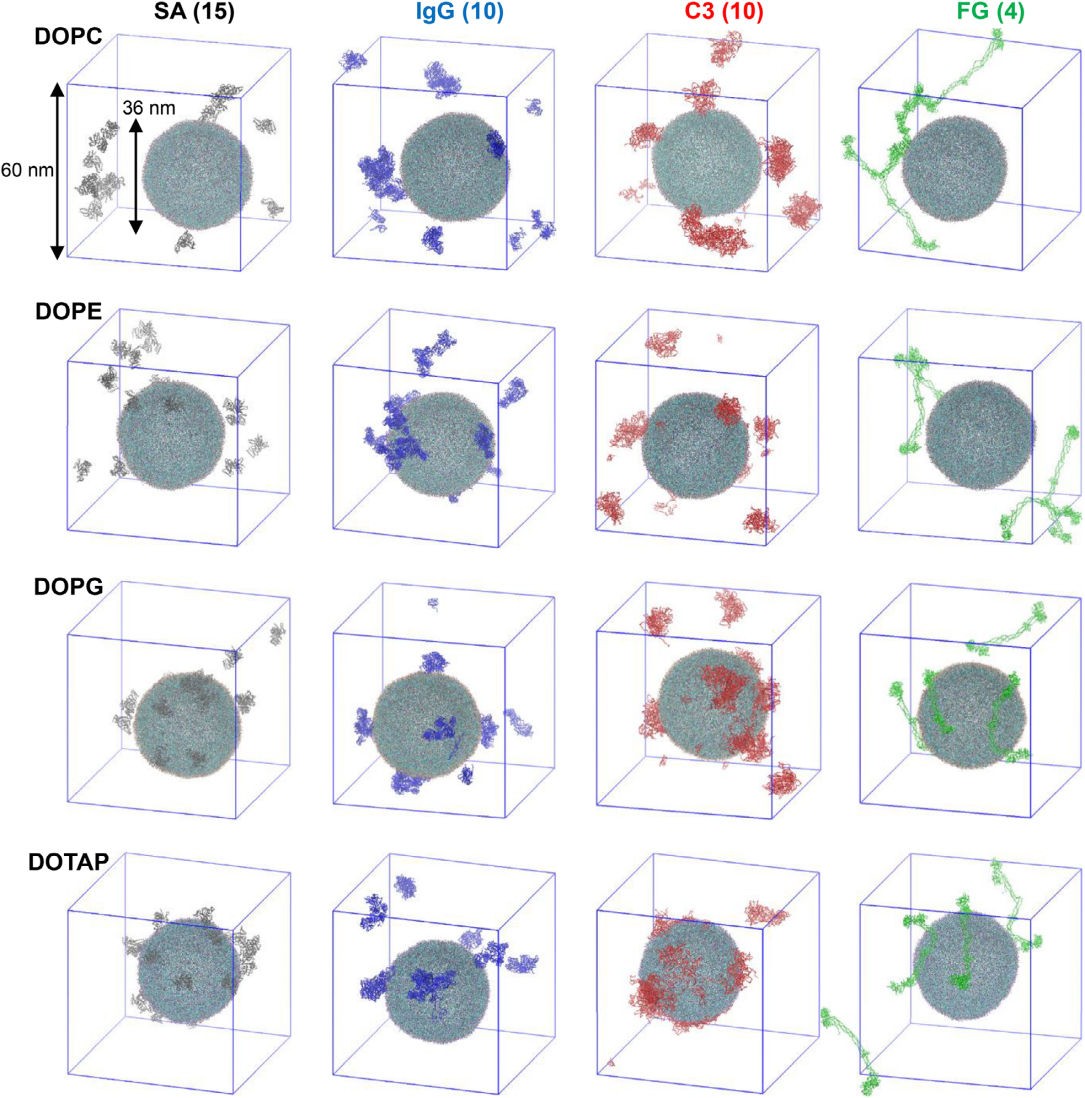
Snapshots at the end (2 µs) of simulations of a 36 nm liposome (DOPC/Chol., DOPE/Chol., DOPG/Chol., and DOTAP/Chol. from the 1^st^ to 4^th^ rows) interacting with plasma proteins such as 15 human serum albumin (SA; grey), 10 immunoglobulin gamma‐1 (IgG; blue), 10 complement C3 (C3; red), or 4 fibrinogen (FG; green) molecules in a cubic periodic box of size 60 nm per side. Water and ions are omitted for clarity.

**FIGURE 3:**
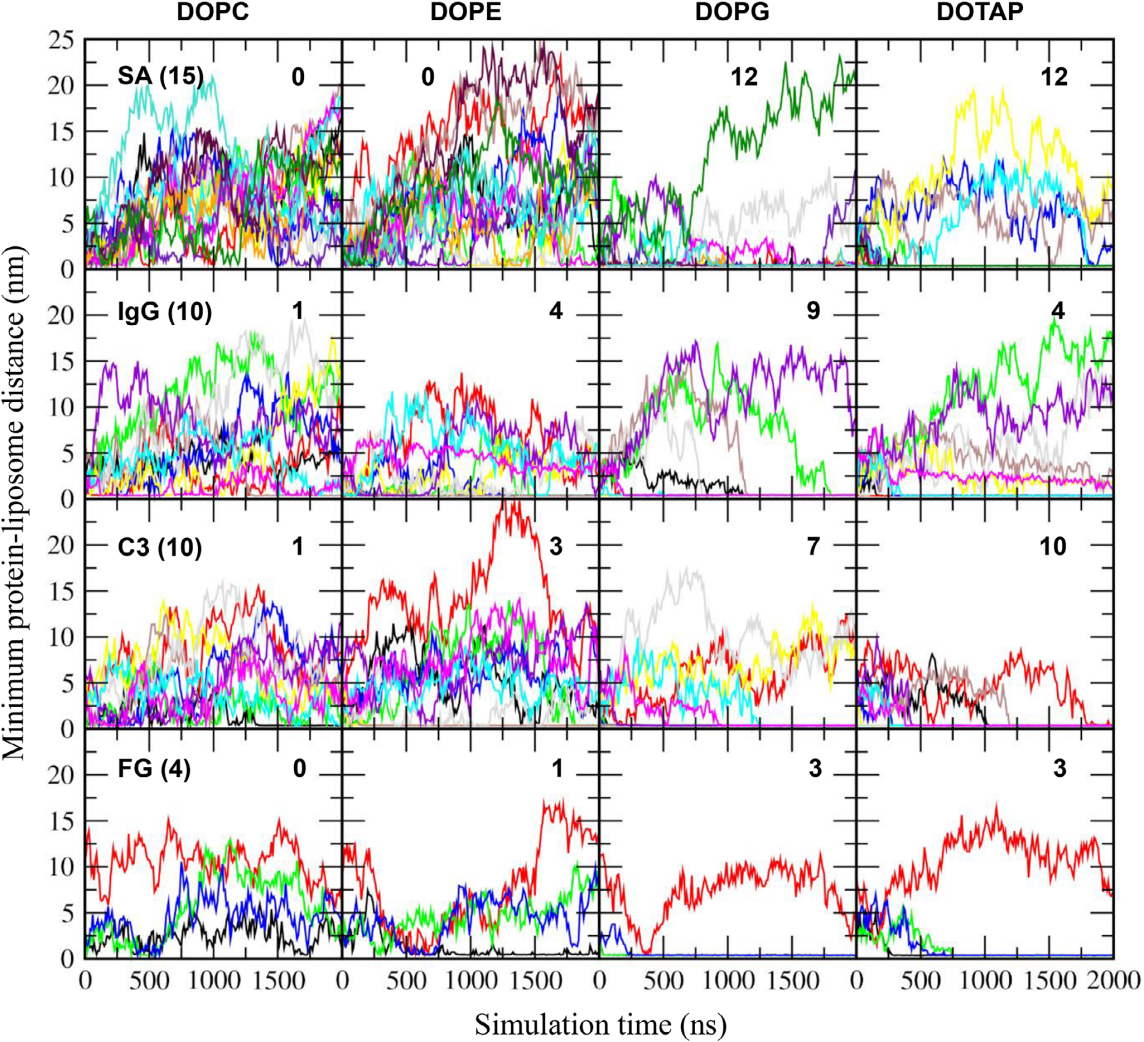
Minimum distances between each protein and a 36 nm liposome as a function of time. Each system contains 15 SA, 10 IgG, 10 C3, or 4 FG proteins (as indicated in parentheses), which are shown in different colors. Numbers of proteins adsorbed to the liposome surface are also indicated.

Single plasma protein species, such as SA, IgG, C3, or FG, were simulated with each equilibrated liposome in water containing 0.15 m NaCl for 2 µs, as listed in Table 1. The initial configurations contained either 15 SA, 10 IgG, 10 C3, or 4 FG molecules, randomly positioned around a 36 nm liposome with a minimum distance of 2 nm between proteins and between proteins and the liposome. In Figure 2, some proteins adsorb to the liposome surface during the simulations, but the extents of adsorption differ. To quantify this, the minimum distances between each protein and the liposome were calculated. In Figure 3, these distances initially fluctuate but in some cases converge to below 0.4 nm, indicating stable binding between proteins and the liposome surface. The number of adsorbed proteins are compared across four liposomes, showing preferential protein adsorption to charged DOPG and DOTAP liposomes rather than to zwitterionic DOPC and DOPE liposomes. For zwitterionic liposomes, protein adsorption is more pronounced on DOPE liposomes, which have smaller amine headgroups, than on DOPC liposomes, which have larger choline headgroups. This observation, although CG models represent donor and acceptor characteristics but cannot explicitly capture hydrogen bonding, is consistent with our previous all‐atom simulations showing that proteins form many more hydrogen bonds with the DOPE bilayer due to its smaller headgroups, as the amine group (NH_3_^+^) of DOPE is well exposed and thus readily binds to proteins, whereas the N^+^ atom of the DOPC choline is sterically hindered by three methyl groups [69].

**TABLE 1:** List of simulations.

	Liposome Size(System)	Number of Molecules									No. of Simul.
		Liposome					Plasma Protein				
		DOPC	DOPE	DOPG	DOTAP	Chol.	SA	IgG	C3	FG	
Single	26 nm	3287	—	—	—	1408	15	—	—	—	1
protein	(50 nm/side)	3287	—	—	—	1408	—	10	—	—	1
species		3287	—	—	—	1408	—	—	10	—	1
		3287	—	—	—	1408	—	—	—	4	1
		—	3564	—	—	1526	15	—	—	—	1
		—	3564	—	—	1526	—	10	—	—	1
		—	3564	—	—	1526	—	—	10	—	1
		—	3564	—	—	1526	—	—	—	4	1
		—	—	3544	—	1519	15	—	—	—	1
		—	—	3544	—	1519	—	10	—	—	1
		—	—	3544	—	1519	—	—	10	—	1
		—	—	3544	—	1519	—	—	—	4	1
		—	—	—	3543	1518	15	—	—	—	1
		—	—	—	3543	1518	—	10	—	—	1
		—	—	—	3543	1518	—	—	10	—	1
		—	—	—	3543	1518	—	—	—	4	1
	36 nm	7706	—	—	—	3302	15	—	—	—	1
	(60 nm/side)	7706	—	—	—	3302	—	10	—	—	1
		7706	—	—	—	3302	—	—	10	—	1
		7706	—	—	—	3302	—	—	—	4	1
		—	7706	—	—	3302	15	—	—	—	1
		—	7706	—	—	3302	—	10	—	—	1
		—	7706	—	—	3302	—	—	10	—	1
		—	7706	—	—	3302	—	—	—	4	1
		—	—	7706	—	3302	15	—	—	—	1
		—	—	7706	—	3302	—	10	—	—	1
		—	—	7706	—	3302	—	—	10	—	1
		—	—	7706	—	3302	—	—	—	4	1
		—	—	—	7706	3302	15	—	—	—	1
		—	—	—	7706	3302	—	10	—	—	1
		—	—	—	7706	3302	—	—	10	—	1
		—	—	—	7706	3302	—	—	—	4	1
Four	36 nm	7706	—	—	—	3302	20	8	2	2	3
protein	(60 nm/side)	—	7706	—	—	3302	20	8	2	2	3
species		—	—	7706	—	3302	20	8	2	2	3
		—	—	—	7706	3302	20	8	2	2	3

**FIGURE 4:**
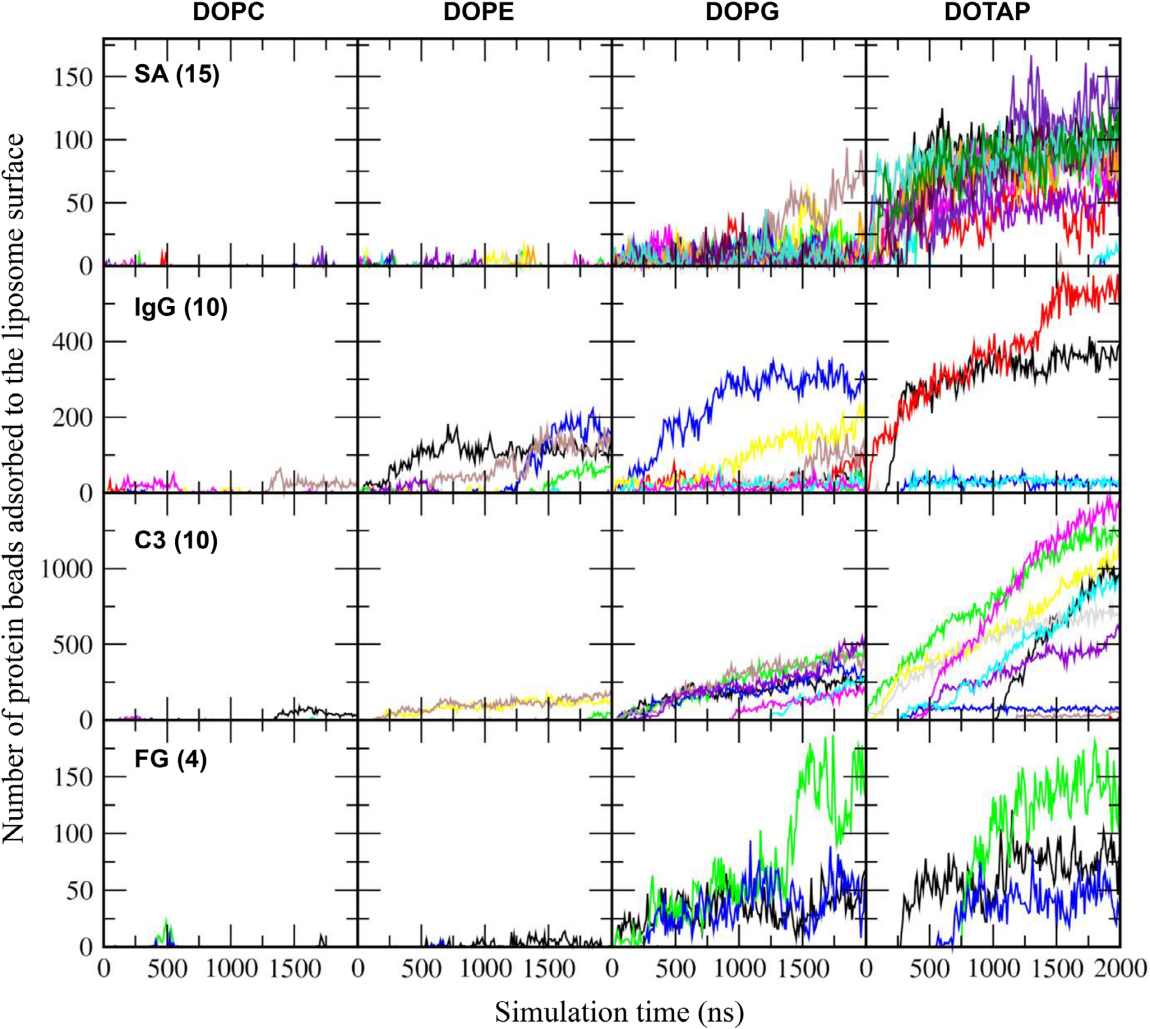
Numbers of protein beads adsorbed to the 36 nm liposome as a function of time. Each system contains 15 SA, 10 IgG, 10 C3, or 4 FG proteins (as indicated in parentheses), which are shown in different colors.

These results were further confirmed by calculating the number of protein beads adsorbed onto the liposome surface, with adsorption defined as any protein bead located within a distance of 0.5 nm from the liposome. In Figure 4, significantly more protein beads adsorb onto charged DOPG and DOTAP liposomes than onto zwitterionic DOPC and DOPE liposomes, consistent with the trends observed in Figure 3 and with experimental results showing greater protein corona formation on charged liposomes [27, 28, 29]. For zwitterionic liposomes, more proteins adsorb onto DOPE liposomes than onto DOPC liposomes, as also observed in Figure 3, again in agreement with experimental findings [29, 30] and our previous simulations [69]. In particular, although both DOPG and DOTAP liposomes are highly charged, many more beads of anionic SA and C3 adsorb onto cationic DOTAP liposomes than onto anionic DOPG liposomes, indicating strong electrostatic interactions between anionic plasma proteins and cationic liposomes, which also agrees with experimental observations showing greater protein adsorption in the order cationic > anionic > neutral liposomes [31].

### 2.3 Electrostatic and Hydrophobic Interactions Between Proteins and Liposomes

**FIGURE 5:**
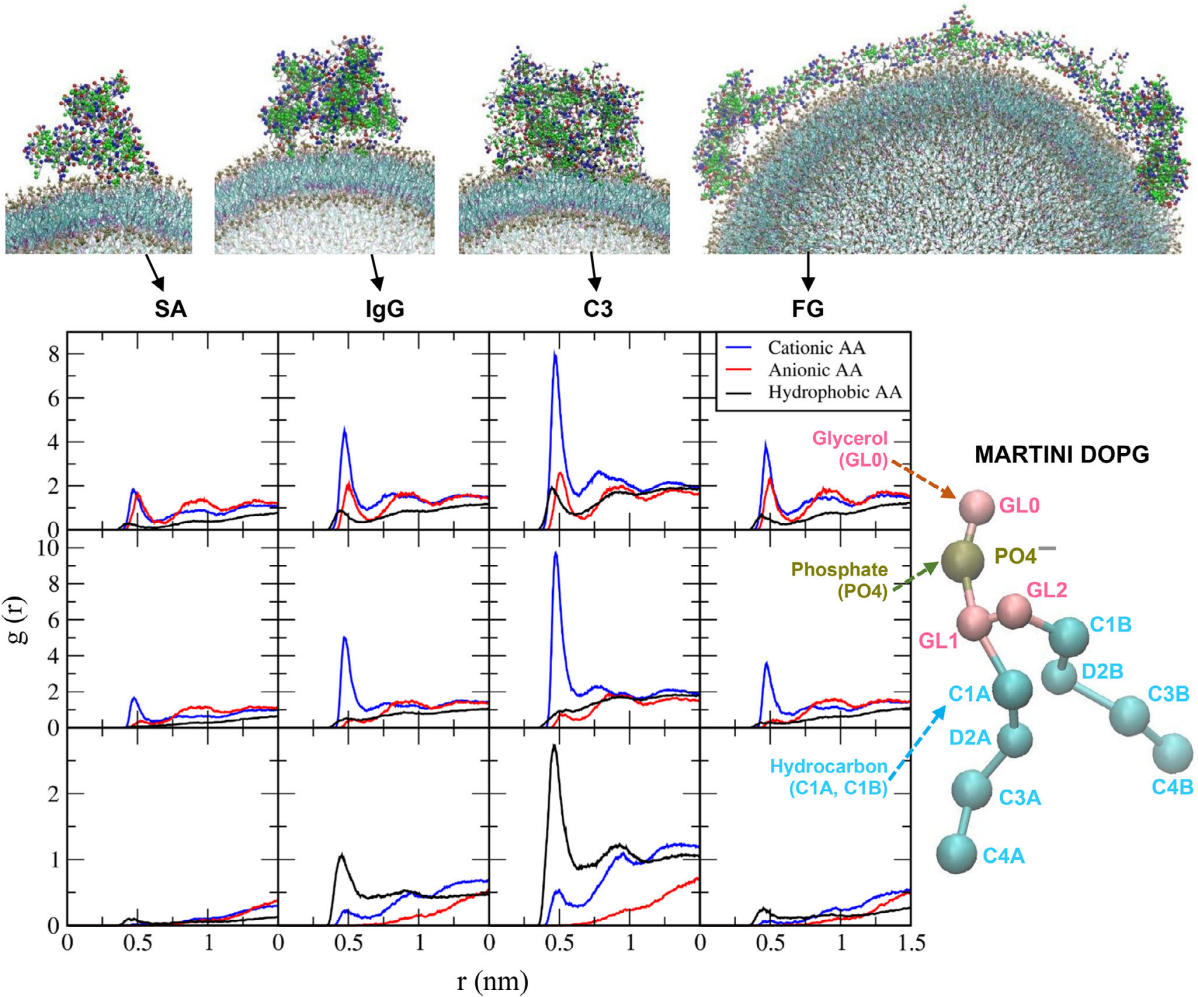
Radial distribution functions (RDFs) of cationic (Arg and Lys), anionic (Asp and Glu), and hydrophobic (Ala, Val, Ile, Leu, Met, Phe, and Trp) amino acids (AAs) in SA, IgG, C3, and FG proteins with respect to the DOPG headgroup (glycerol and phosphate) and hydrocarbon tail. In the snapshots, cationic, anionic, and hydrophobic AAs and anionic DOPG phosphate beads are shown as blue, red, green, and brown dots, respectively.

**FIGURE 6:**
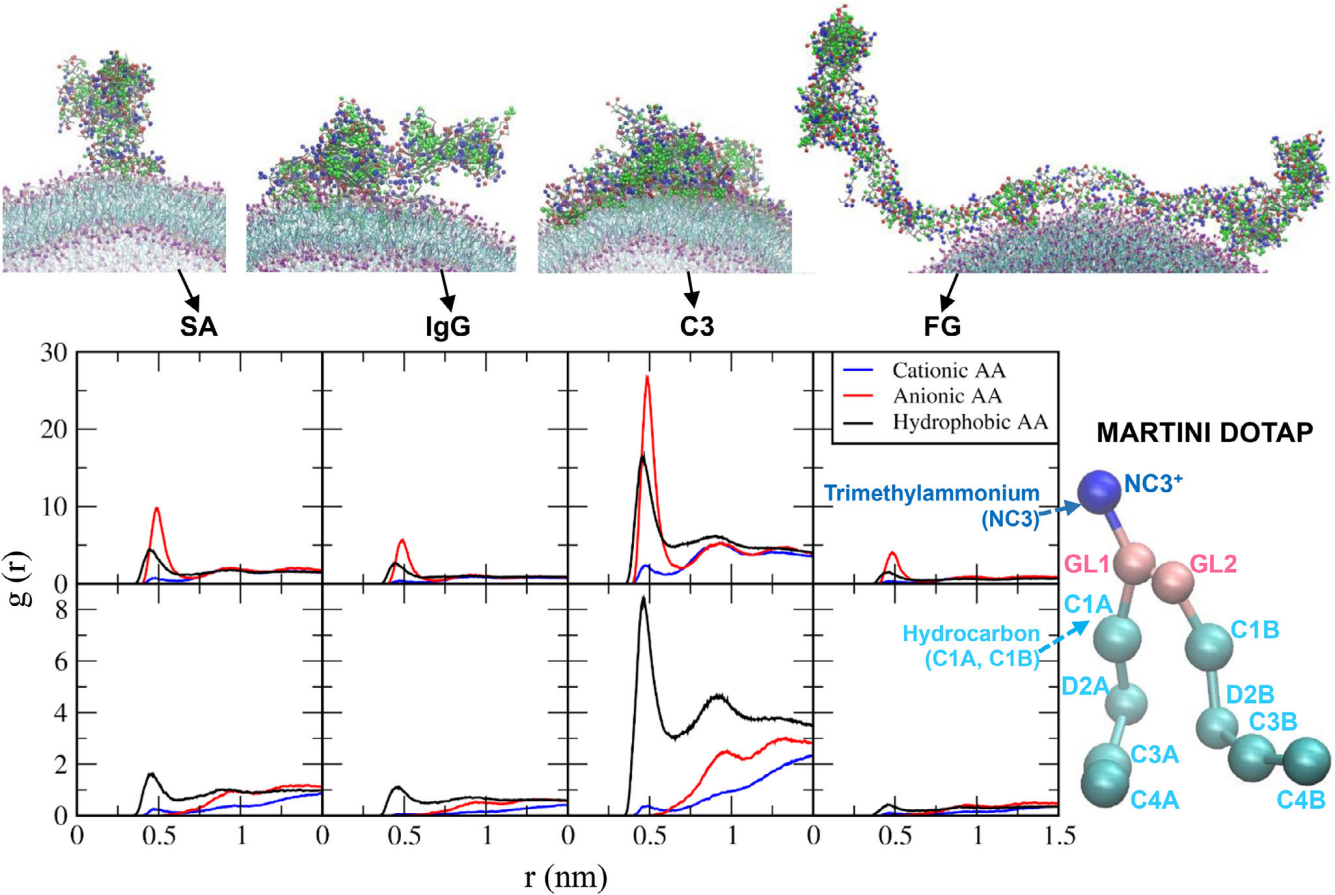
RDFs of cationic, anionic, and hydrophobic AAs in SA, IgG, C3, and FG proteins with respect to the head group (NC3) and the tail group (C1A and C1B) of DOTAP lipids. In the snapshots, cationic, anionic, and hydrophobic AAs and cationic DOTAP trimethylammonium beads are shown as blue, red, green, and purple dots, respectively.

To understand liposome‐protein interactions, radial distribution functions (RDFs) were calculated for cationic (Arg and Lys), anionic (Asp and Glu), and hydrophobic (Ala, Val, Ile, Leu, Met, Phe, and Trp) amino acids of proteins with respect to lipid headgroups (glycerol and phosphate in DOPG; trimethylammonium in DOTAP) and hydrocarbon tails. In Figure 5, RDF peaks are higher between charged amino acids and DOPG headgroups, and between hydrophobic amino acids and DOPG tails, indicating electrostatic interactions with the lipid headgroups and hydrophobic interactions with the lipid tails. In particular, the RDF peaks of the anionic DOPG headgroups are much higher for cationic amino acids than for anionic amino acids, indicating strong electrostatic interactions between cationic residues and anionic DOPG headgroups. Figure 6 shows the RDF peaks for cationic, anionic, and hydrophobic amino acids with respect to the DOTAP headgroups and hydrocarbon tails. The RDF peaks of the cationic DOTAP headgroups are much higher for anionic amino acids than for cationic amino acids, as expected, indicating strong electrostatic interactions. High RDF peaks are also observed between hydrophobic amino acids and DOTAP tails, indicating hydrophobic interactions. In particular, for SA and C3 proteins, the RDF peaks between anionic amino acids and cationic DOTAP headgroups are much higher than those between cationic amino acids and anionic DOPG headgroups, consistent with Figure 4 showing greater adsorption of anionic SA and C3 proteins onto cationic DOTAP liposomes than onto anionic DOPG liposomes. These results indicate that plasma proteins adsorb onto both cationic and anionic liposomes but bind more strongly to oppositely charged liposomes via electrostatic and hydrophobic interactions, implying the dependence of liposome‐protein interactions on both protein and lipid charge.

### 2.4 Effect of Liposome Size on Protein Adsorption

**FIGURE 7:**
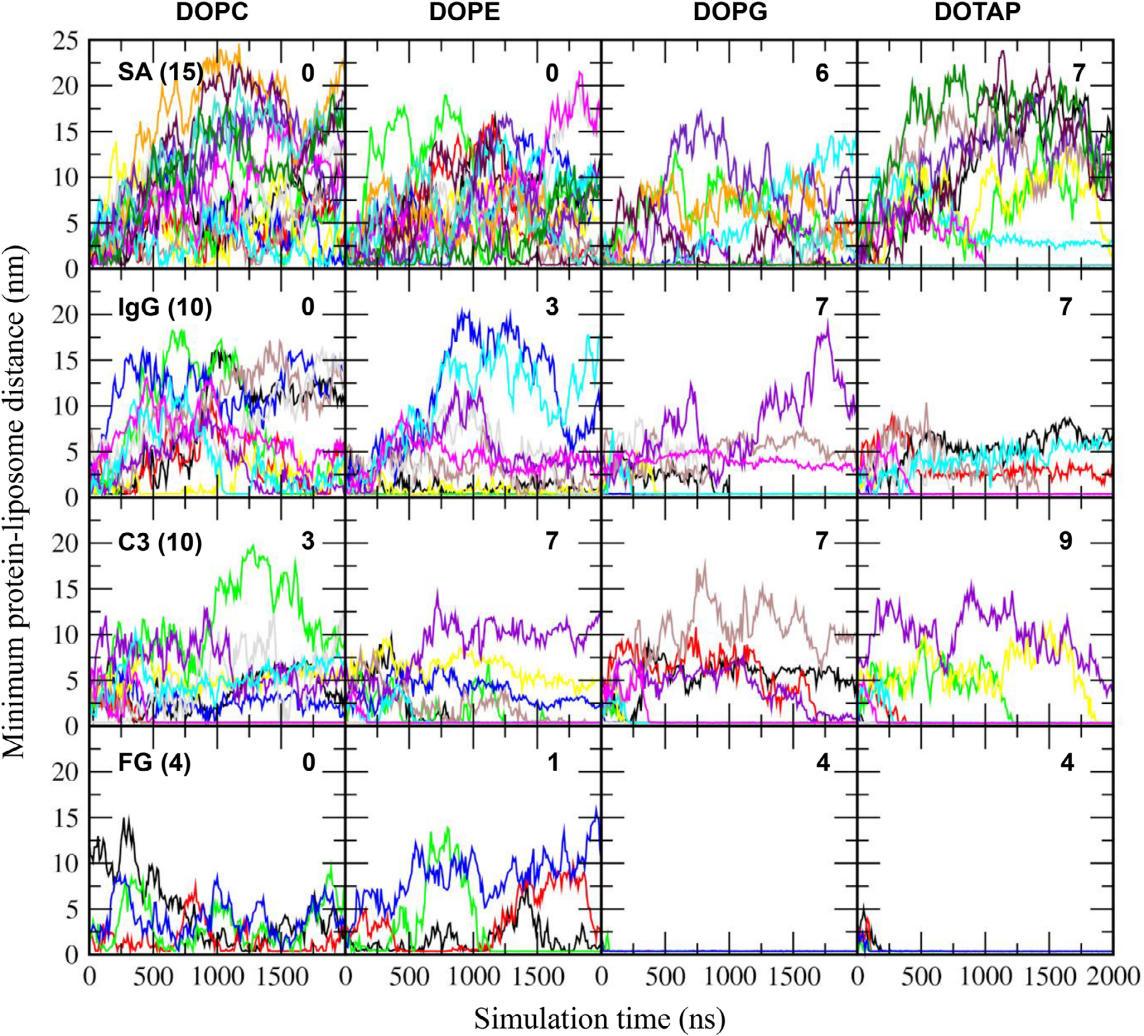
Minimum distances between each protein and a 26 nm‐sized liposome as a function of time. Each system contains 15 SA, 10 IgG, 10 C3, or 4 FG proteins (as indicated in parentheses), which are shown in different colors. Numbers of proteins adsorbed to the liposome surface are also indicated.

**FIGURE 8:**
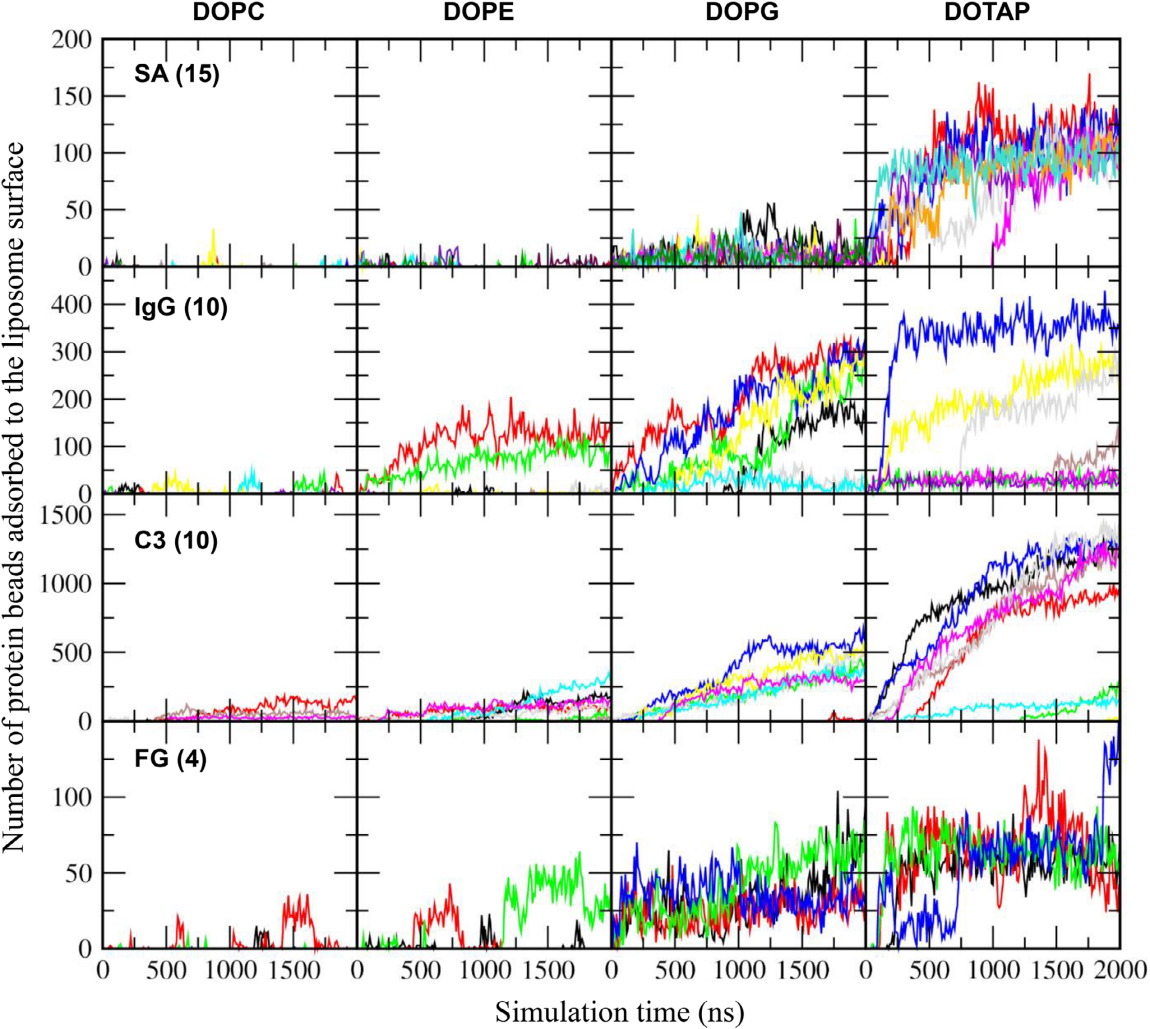
Numbers of protein beads adsorbed to the 26 nm liposome as a function of time. Each system contains 15 SA, 10 IgG, 10 C3, or 4 FG proteins (as indicated in parentheses), which are shown in different colors.

Previous experimental and simulation studies have shown that particle size (i.e., curvature) influences protein adsorption, although most have been performed with carbon nanotube, gold, or polystyrene nanoparticle surfaces [37, 40, 74, 75, 76], which differ from the surface properties of zwitterionic or charged liposomes. To understand the effect of liposome size on protein adsorption, smaller 26 nm liposomes composed of cholesterol and phospholipids (DOPC, DOPE, DOPG, or DOTAP) were also simulated with either 15 SA, 10 IgG, 10 C3, or 4 FG proteins, similar to the simulations with the 36 nm liposome. Minimum distances between each protein and the liposome were calculated, which show that some values converge to below 0.4 nm (Figure 7), indicating protein adsorption onto the liposome surface. This adsorption occurs predominantly for charged liposomes rather than neutral ones. For neutral liposomes, a greater number of proteins adsorb to DOPE liposomes than to DOPC liposomes. These are consistent with the trends observed for the 36 nm liposome, which were also confirmed by calculating the number of protein beads adsorbed to the 26 nm liposome (Figure 8).

**FIGURE 9:**
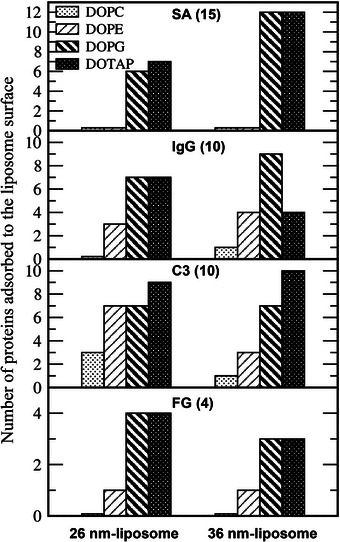
Numbers of plasma proteins adsorbed to the 26 and 36 nm‐sized liposomes composed of DOPC, DOPE, DOPG, and DOTAP.

**FIGURE 10:**
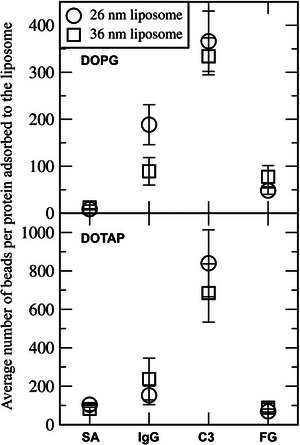
Average number of beads per protein adsorbed to the liposome composed of DOPG or DOTAP.

To further examine the effect of liposome size on protein adsorption, Figure 9 compares the number of proteins adsorbed onto 26 and 36 nm liposomes, again confirming that more proteins adsorb onto charged liposomes and liposomes with smaller headgroups, regardless of liposome size. In particular, many fewer SA proteins, which are heart‐shaped molecules with dimensions of 8 × 8 × 3 nm^3^ and the smallest of the four proteins simulated, bind to 26 nm charged liposomes than to 36 nm charged liposomes, indicating reduced SA adsorption on smaller liposomes, similar to experimental observations and simulations showing weaker SA adsorption on more curved surfaces of carbon nanotubes and gold nanoparticles [36, 37, 74, 75]. Note that the surface area of a 36 nm liposome is approximately 1.9 times that of a 26 nm liposome, and this ratio is consistent with the trend in the amount of adsorbed SA on charged liposomes. This implies that the protein adsorption density per unit area remains approximately constant and that the overall extent of SA adsorption seems to be similar for 26 and 36 nm liposomes. However, this consistency is observed only for SA and not for IgG, C3, or FG, whose adsorbed amounts do not change significantly between 26 and 36 nm liposomes, indicating that the difference in SA adsorption is attributed to changes in liposome curvature rather than differences in available surface area. In addition, Figure 10 compares the average number of beads per adsorbed protein, which shows much lower values for SA than for larger proteins such as IgG and C3, indicating that individual SA proteins interact less extensively with the liposome surface. This implies that SA adsorption can be more readily reduced by increased bilayer curvature (i.e., smaller liposome size) than the adsorption of larger proteins, although it cannot be ruled out that liposomes larger than the simulated 36 nm may have a greater influence on the adsorption of larger proteins. For cell membranes with almost no curvature, previous experiments and simulations have shown that the adsorption of proteins (protein corona) on drug carriers can either promote the cellular uptake and internalization of drug carriers via cell surface receptors [77] or sterically block carrier‐membrane binding and thus suppress their adsorption onto and internalization into cell membranes, whose extracellular leaflet mainly consists of neutral zwitterionic lipids [68, 78].

### 2.5 Competitive Adsorption of Four Protein Species Onto the Liposome Surface

To more realistically mimic competitive protein adsorption in plasma environments, a mixture of SA, IgG, C3, and FG proteins was simulated with a 36 nm liposome composed of cholesterol and phospholipids such as DOPC, DOPE, DOPG, or DOTAP for 2 µs, as listed in Table 1. Considering the relative abundances of SA and IgG in human plasma [13, 79], 20 SA, 8 IgG, 2 C3, and 2 FG proteins were randomly placed around the liposome with a minimum distance of 2 nm between proteins and between proteins and the liposome. Three independent simulations with different initial configurations were performed for each system to obtain more samples.

**FIGURE 11:**
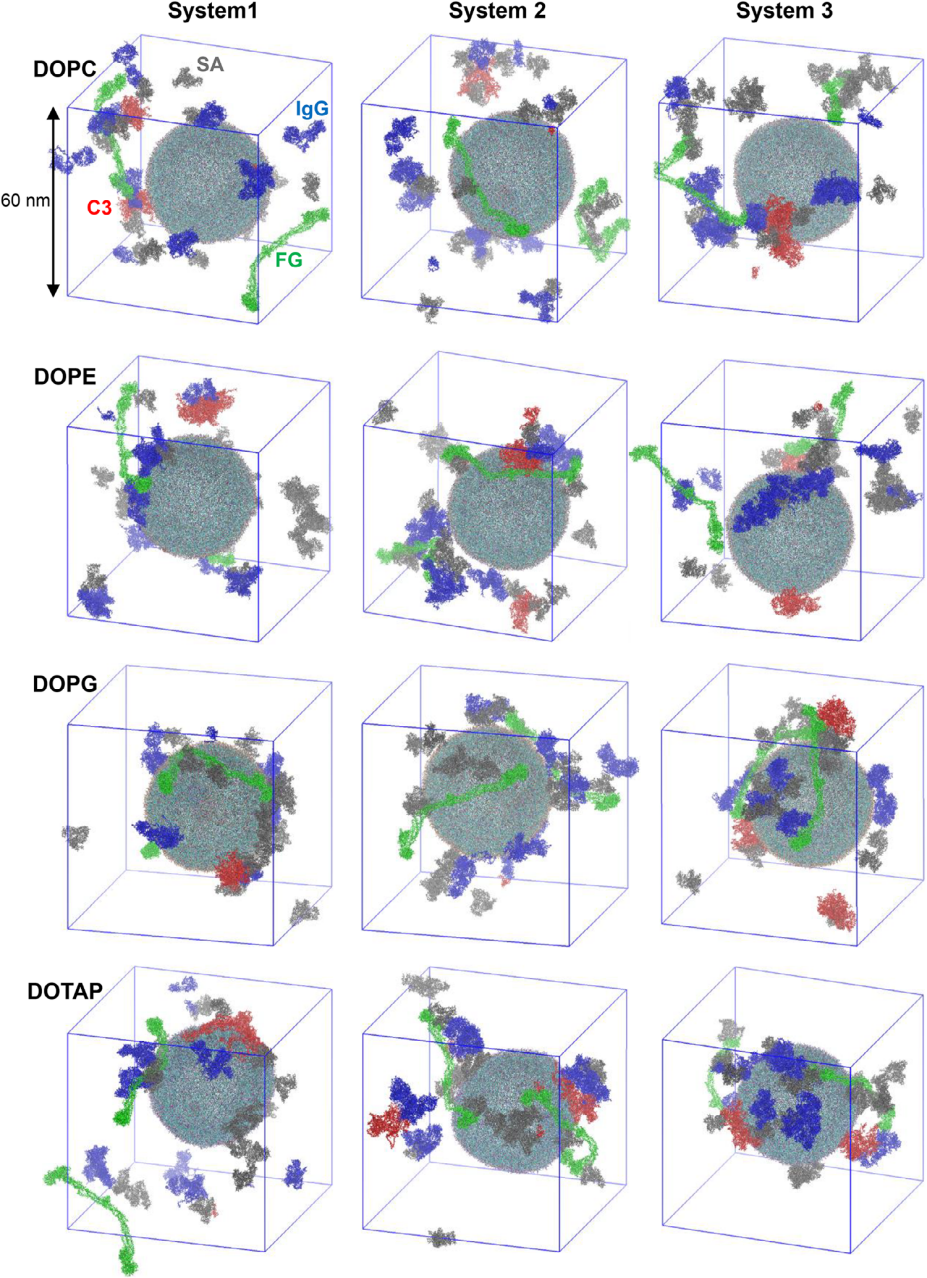
Snapshots at the end (2 µs) of simulations of a 36 nm‐sized liposome (DOPC/Chol., DOPE/Chol., DOPG/Chol., and DOTAP/Chol. from the 1^st^ to 4^th^ rows) interacting with a mixture of 20 SA (grey), 8 IgG (blue), 2 C3 (red), and 2 FG (green) proteins in a cubic periodic box of size 60 nm per side. To improve sampling, three different initial protein configurations were simulated, and hence three snapshots (systems 1, 2, and 3) are shown for each liposome.

**FIGURE 12:**
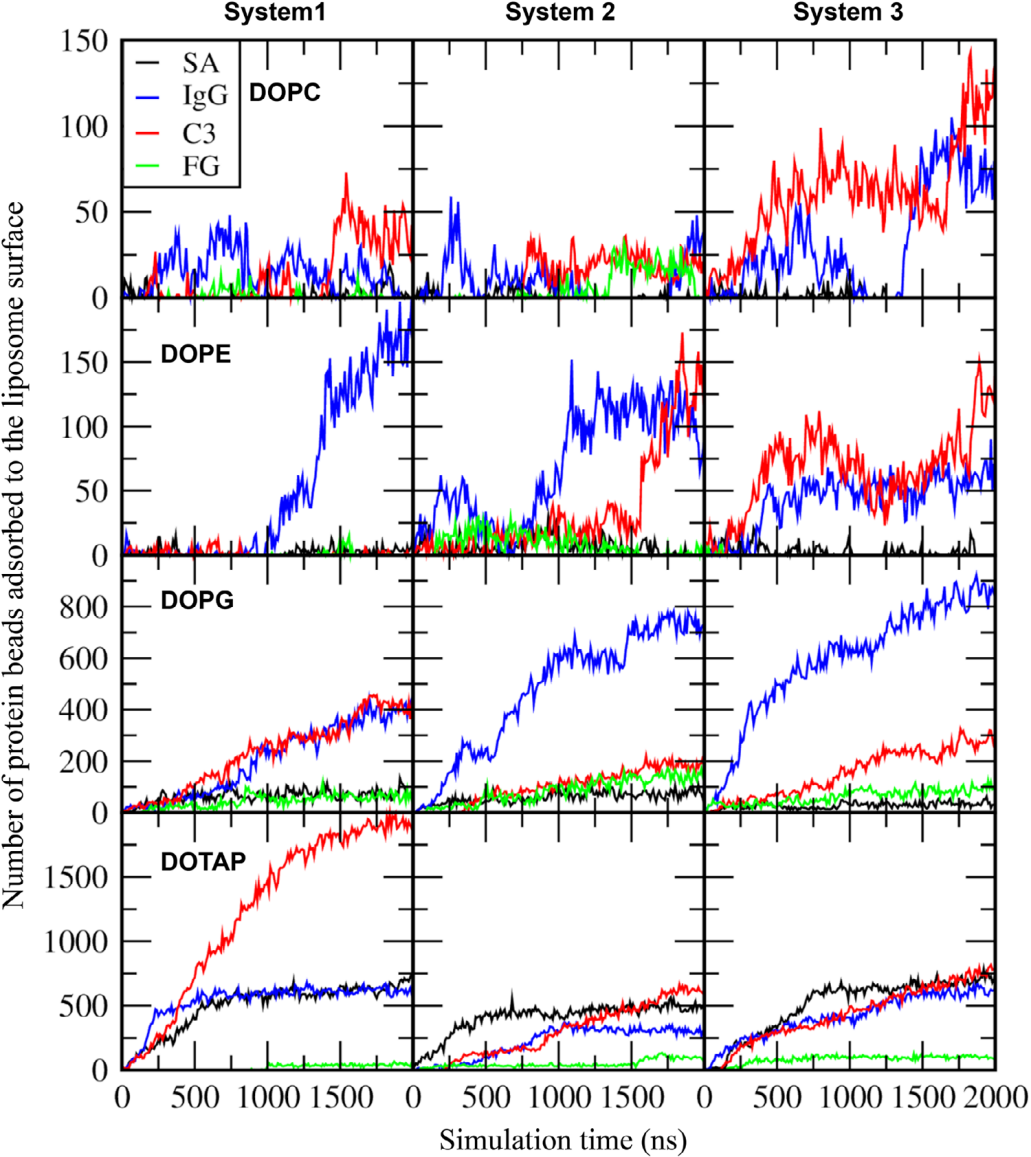
Numbers of protein beads adsorbed to the liposome as a function of time.

**FIGURE 13:**
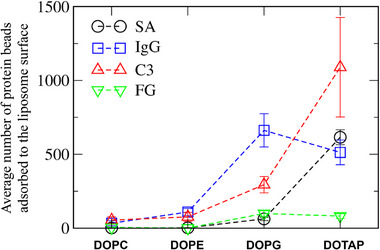
Average number of protein beads adsorbed to the liposome surface, averaged over three independent simulations for each liposome.

Figure 11 shows that some proteins adsorb to the liposome surface, to an extent dependent on liposome composition. To quantify this, we calculated the number of protein beads adsorbed onto the liposome surface. In Figure 12, although each system contains 20 SA proteins, many more than 8 IgG or 2 C3 proteins, IgG and C3 exhibit stronger adsorption to the liposome. FG proteins also adsorb, but to a lesser extent, presumably because of their highly anisotropic shape (6 × 9 × 45 nm^3^) that limits the surface area available for interaction with the liposome. Figure 13 compares the average number of protein beads adsorbed to the liposome surface, averaged over three independent simulations for each liposome. Protein adsorption is higher for charged liposomes than for zwitterionic liposomes, with the highest adsorption observed for cationic DOTAP. Among the neutral liposomes, DOPE, which has a smaller headgroup, shows greater protein adsorption than DOPC. These results agree with experiments [27, 28, 29, 30, 31]. In particular, anionic DOPG exhibits stronger adsorption of cationic IgG (net charge of +19) than of other proteins, while cationic DOTAP shows stronger adsorption of anionic C3 (net charge of −25) than of other proteins, indicating the role of electrostatic interactions. SA (net charge of ‐15) adsorbs more significantly to DOTAP than to other liposomes, which also agrees with experiments showing stronger binding of SA to DOTAP liposomes or lipids [34, 35].

**FIGURE 14:**
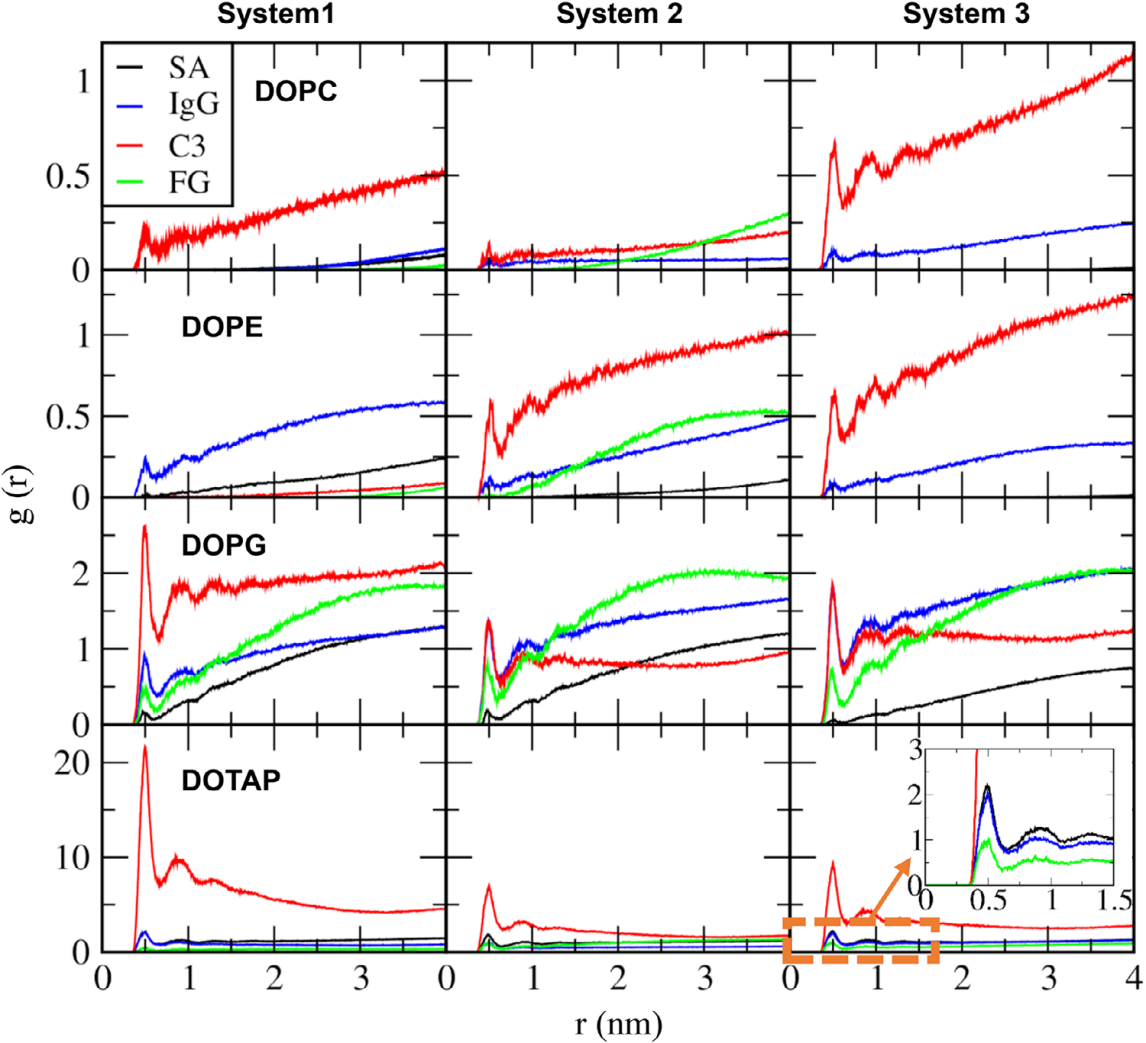
RDFs of SA, IgG, C3, and FG proteins with respect to the lipid headgroups (cationic trimethylammonium bead for DOTAP and anionic phosphate bead for DOPC, DOPE, and DOPG).

To understand these differences in protein adsorption among various liposomes, RDFs between proteins and lipid headgroups were calculated. In Figure 14, no prominent RDF peaks are observed for zwitterionic DOPC and DOPE, whereas high RDF peaks of IgG and C3 are observed for charged DOPG and DOTAP, with particularly high peaks between anionic C3 and cationic DOTAP lipids. RDF peaks of SA are significant only for DOTAP, confirming strong electrostatic interactions between anionic SA and cationic DOTAP lipids. These results show that anionic SA and C3 predominantly adsorb to cationic DOTAP liposomes, whereas cationic IgG preferentially adsorb to anionic DOPG liposomes, indicating the dependence on both liposome and protein electrostatics.

### 2.6 Replacement of Abundant Proteins by High‐Affinity Proteins

Experiments have shown that abundant plasma proteins initially adsorb to the particle surface in human plasma but are later replaced by high‐affinity proteins (the Vroman effect) [80]. This protein replacement has been complemented by theoretical studies and simulations, although most simulations have been performed with implicit solvent and simplified models that cannot explicitly distinguish individual amino acids and lipid headgroups [46, 70], thereby limiting mechanistic understanding of the Vroman effect at nearly atomic resolution. To resolve this, the replacement of abundant proteins (SA) with high‐affinity proteins (IgG or C3) was captured and analyzed in our CG simulations that distinguish individual amino acids and lipid headgroups.

**FIGURE 15:**
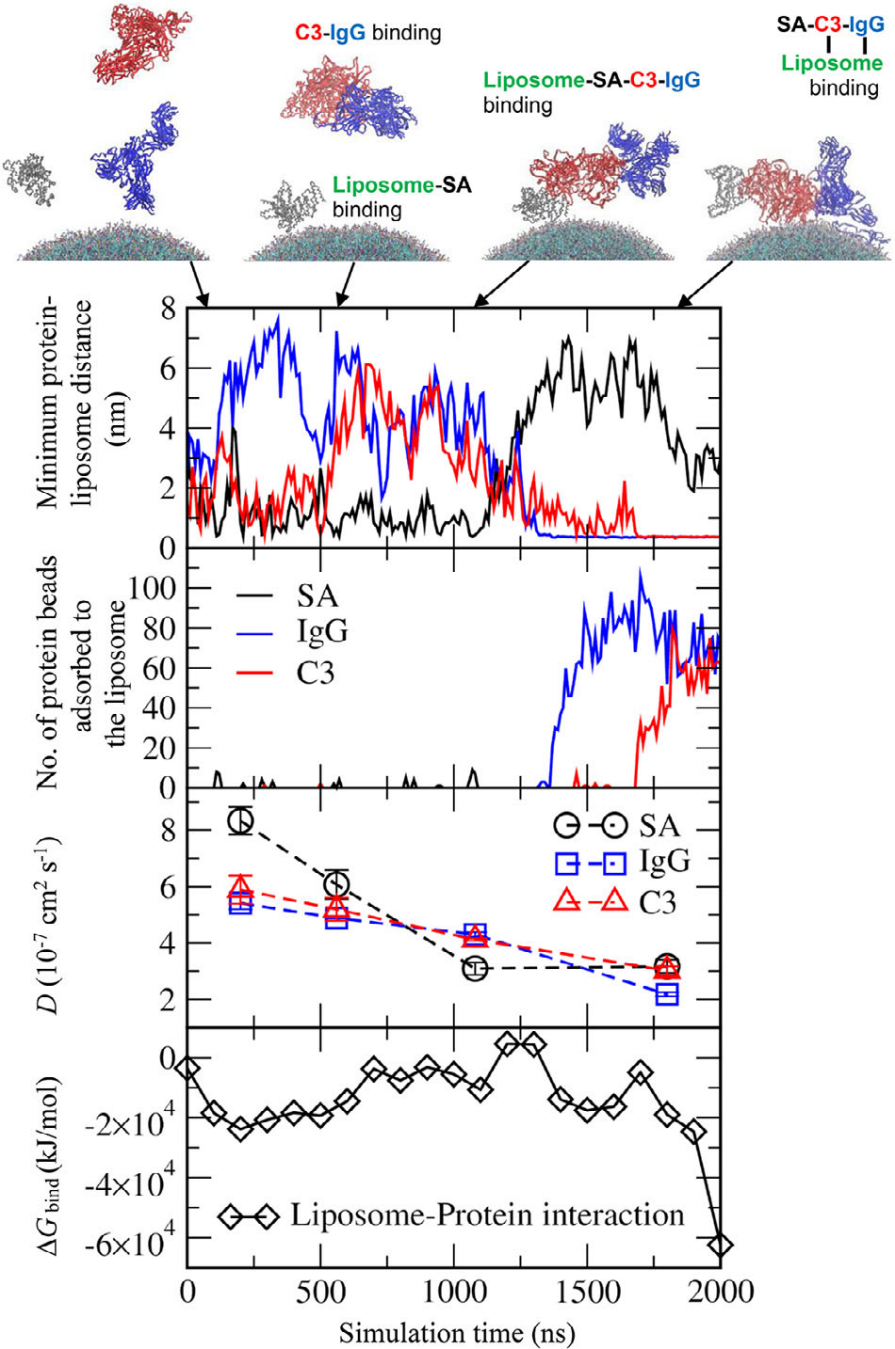
Minimum distances between each protein and a DOPC liposome (top), numbers of protein beads adsorbed to the liposome (2^nd^ row), diffusion coefficients (*D*) of proteins (3^rd^ row), and binding free energies (Δ*G*_bind_) of liposome‐protein interactions (bottom) as a function of time. Interactions of SA (grey), IgG (blue), and C3 (red) proteins with each other and with a DOPC liposome are described in the snapshots.

Figure 15 shows the replacement of SA by IgG and C3 on the DOPC liposome surface as a function of simulation time. Minimum distances between each protein and the liposome were calculated (Figure 15, top), showing that SA adsorbs to the liposome surface at around 600 ns, while C3 and IgG bind together to form a complex. Within this C3‐IgG complex, C3 interacts with SA on the liposome surface, after which SA begins to detach at approximately 1200 ns. Following SA detachment, IgG adsorbs to the liposome surface at around 1300 ns, followed by C3 at about 1700 ns. To quantify the extent of adsorption, the number of protein beads adsorbed onto the liposome was calculated (Figure 15, 2^nd^ graph). The values are much lower for SA than for IgG and C3, indicating that abundant SA binds only weakly and thus can readily detach from the liposome surface. To confirm this binding strength, diffusion coefficients (*D*) of proteins were calculated from the slope of their mean‐square displacement, with finite‐size effect correction (Figure 15, 3^rd^ graph), as described in the Experimental section. When SA adsorbs to the liposome surface, its *D* value decreases, and when the adsorbed SA binds to the C3‐IgG complex, its *D* value decreases even more significantly. This reduced protein diffusivity indicates strong attractive interactions between proteins. Similarly, the *D* values of C3 and IgG also decrease when these proteins bind to other proteins or the liposome surface, showing lower diffusivities upon adsorption or complex formation.

**FIGURE 16:**
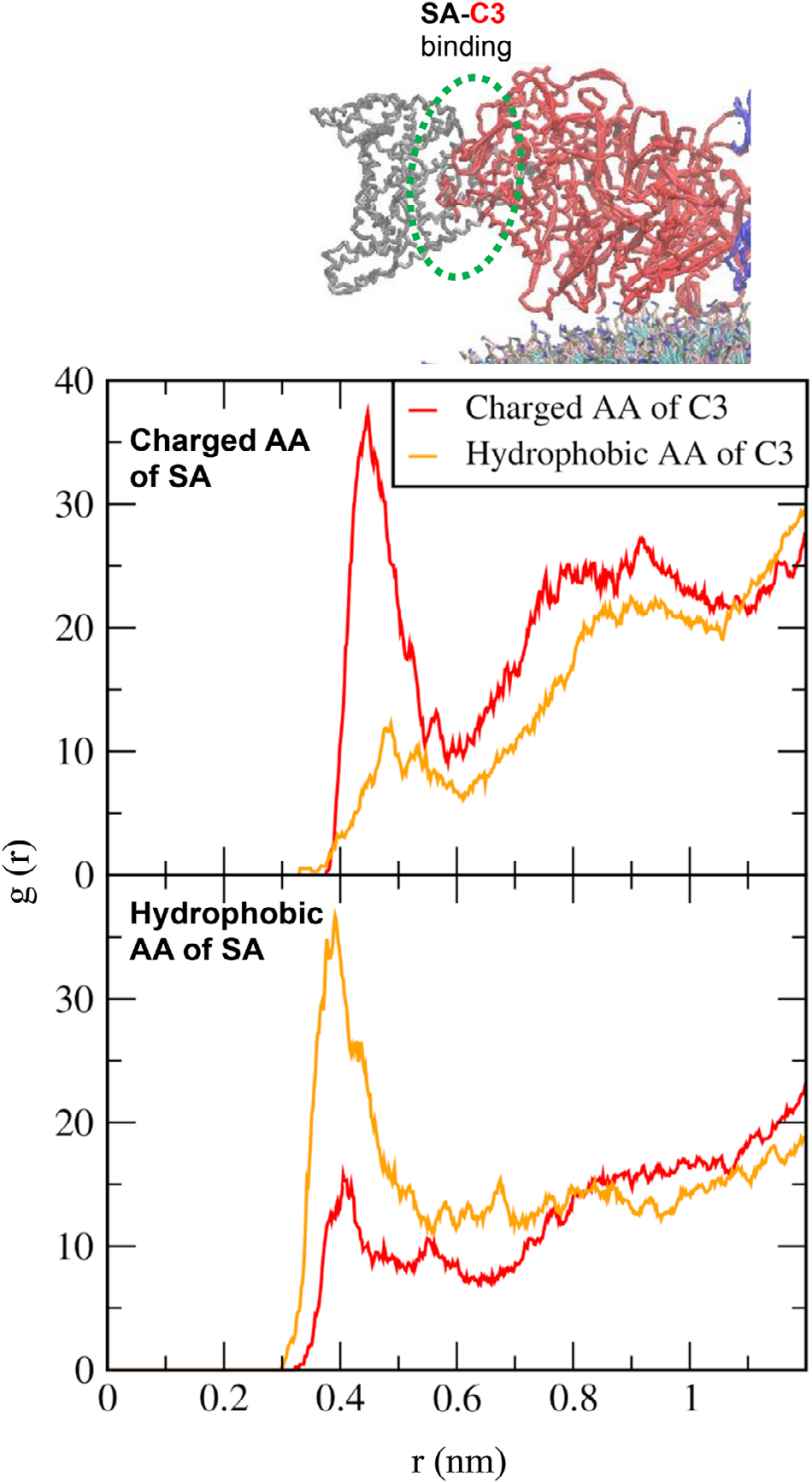
RDFs between charged and hydrophobic AAs of SA and C3 proteins on the DOPC liposome.

To interpret this process of protein replacement on the liposome surface, binding free energies (Δ*G*_bind_) of liposome‐protein interactions were calculated using the molecular mechanics Poisson–Boltzmann surface area (MM‐PBSA) method (Figure 15, bottom) [81, 82], as described in the Experimental section. When adsorbed SA detaches from the liposome surface, and IgG begins to bind (around 1300 ns), Δ*G*_bind_ decreases significantly, and it decreases even further after C3 also binds to the liposome surface (around 1700 ns), indicating that liposome‐protein interactions are energetically stabilized by the replacement of abundant SA with high‐affinity C3 and IgG, supporting the Vroman effect [80]. To understand the protein‐protein interaction to promote this replacement, RDFs between charged and hydrophobic amino acids of SA and C3 were calculated. In Figure 16, high RDF peaks are observed between charged residues of SA and C3, as well as between their hydrophobic residues, indicating strong SA‐C3 interactions mediated by both electrostatic and hydrophobic forces. These diffusivity, free energy, and RDF calculations imply that the replacement of SA by C3 and IgG on the liposome surface is due to the weaker binding of SA to the liposome surface compared with C3 and IgG, as well as the strong electrostatic and hydrophobic protein‐protein (SA‐C3 or SA‐IgG) interactions.

**FIGURE 17:**
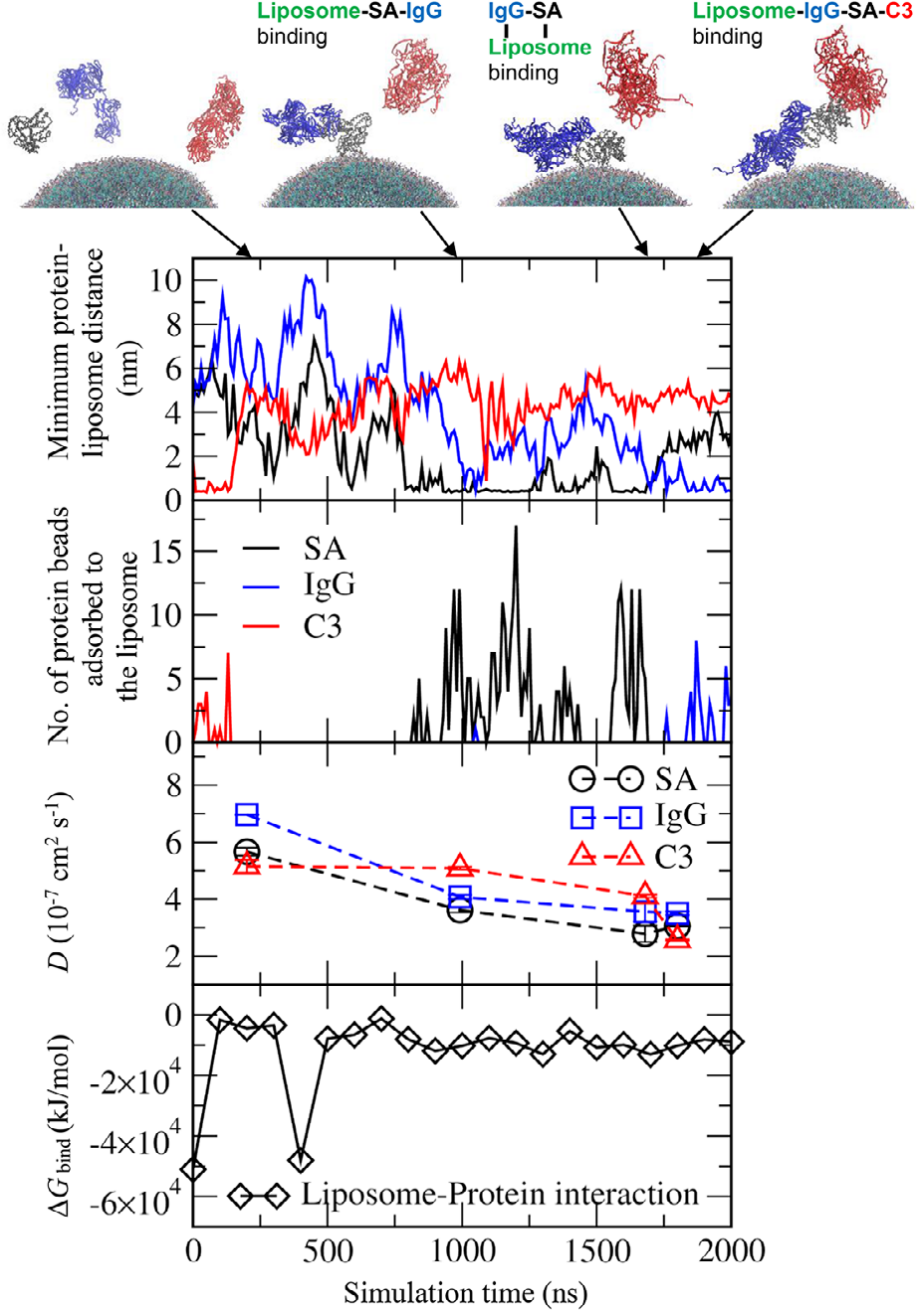
Minimum distances between each protein and a DOPE liposome (top), numbers of protein beads adsorbed to the liposome (2^nd^ row), diffusion coefficients (*D*) of proteins (3^rd^ row), and binding free energies (Δ*G*_bind_) of liposome‐protein interactions (bottom) as a function of time. Interactions of SA (grey), IgG (blue), and C3 (red) proteins with each other and with a DOPE liposome are described in the snapshots.

**FIGURE 18:**
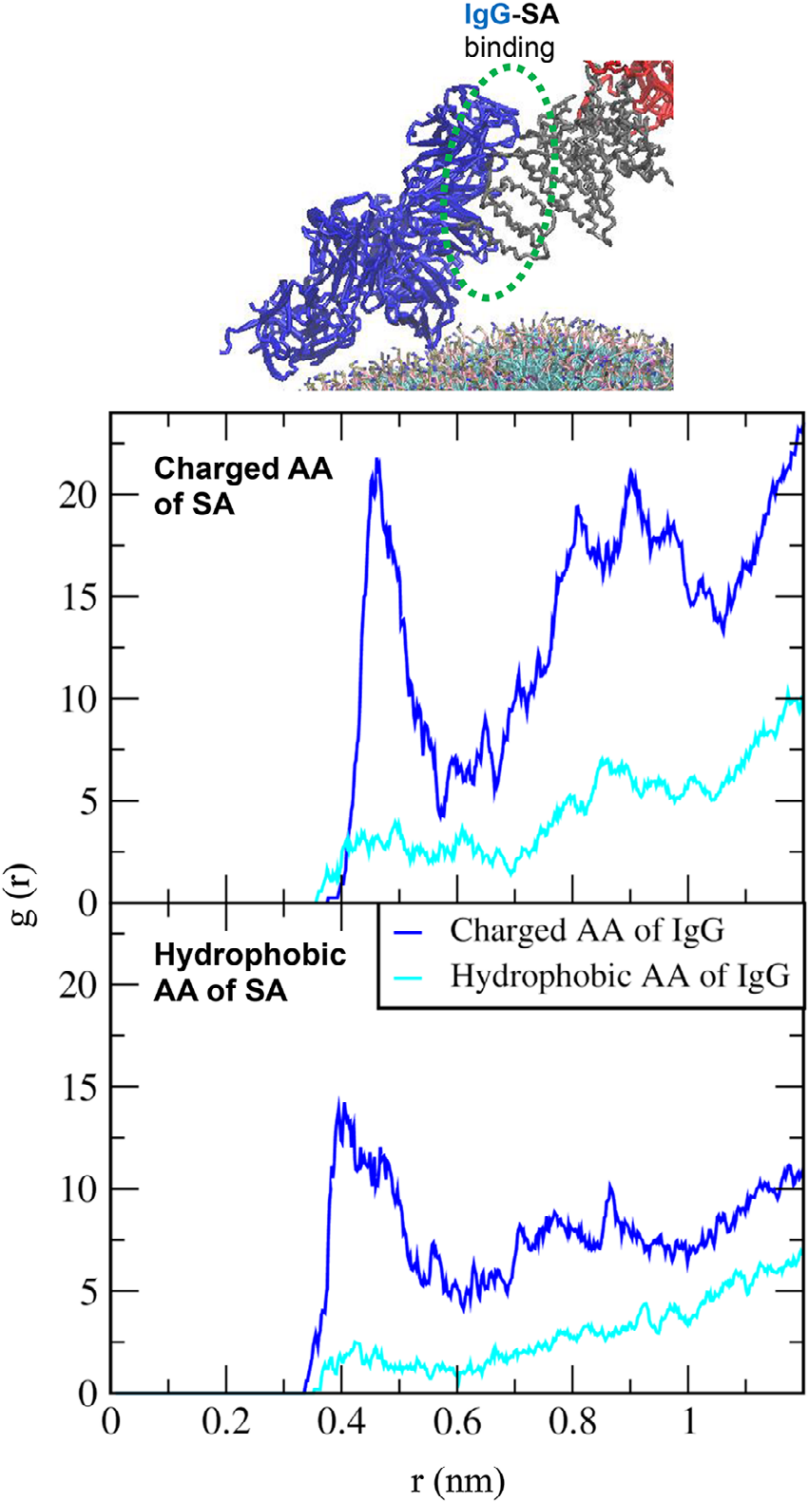
RDFs between charged and hydrophobic AAs of SA and IgG proteins on the DOPE liposome.

Similar to the protein replacement observed on the DOPC liposome, the replacement of SA by IgG on the DOPE liposome surface was also analyzed. Figure 17 shows that SA binds to the liposome around 800 ns, and the adsorbed SA subsequently binds to IgG at approximately 1000 ns, forming the SA‐IgG complex. IgG then begins to bind to the liposome surface around 1700 ns, while SA simultaneously starts to detach, showing the replacement of SA by IgG on the liposome surface. Protein diffusivities decrease when SA and IgG form the complex and when they individually bind to the liposome surface, again confirming protein‐protein and protein‐liposome interactions. Note that when adsorbed SA is replaced by IgG on the DOPE liposome surface, Δ*G*_bind_ shows little change (in contrast to the drastic decrease observed when SA is replaced by IgG and C3 on the DOPC liposome), presumably because of the weaker interactions of proteins with DOPE compared to DOPC in this specific case (Figures 15 and 17, 2^nd^ graphs). In Figure 18, RDFs are much higher for charged residues than for hydrophobic residues, indicating that the binding between anionic SA and cationic IgG is attributed to electrostatic interactions rather than hydrophobic interactions, in contrast to SA‐C3 binding (Figure 16), indicating the dependence of protein‐protein binding on protein type.

Experiments have shown greater adsorption of plasma proteins onto charged liposomes than onto neutral liposomes, with particularly high adsorption onto cationic liposomes [27, 28, 29, 31]. For neutral liposomes, those with smaller lipid headgroups exhibit greater protein adsorption. [29, 30] Also, less protein adsorption was observed on more highly curved nanoparticle surfaces [36, 37], although the surface properties of these nanoparticles differ from those of liposomes. These experimental results agree qualitatively with our simulations of four major plasma proteins adsorbed onto 26 and 36 nm‐sized liposomes. Simulations show that the preferential adsorption of plasma proteins onto charged liposomes is attributed to electrostatic interactions with lipid headgroups and hydrophobic interactions with lipid tails, depending on both liposome and protein electrostatics. Because SA binds relatively weakly, it is more easily detached from the liposome surface at higher bilayer curvature, resulting in reduced adsorption on smaller liposomes. In particular, diffusivity and binding free‐energy calculations show that liposome‐protein interactions are energetically stabilized by replacing abundant SA by high‐affinity IgG or C3 through electrostatic and hydrophobic interactions between proteins and those between proteins and lipids. These results, combined with our previous all‐atom simulations of plasma proteins adsorbed onto lipid bilayers with different charges [69], suggest that liposomes composed of zwitterionic lipids, in which the nitrogen atom in the headgroup is less accessible, rather than charged lipids or zwitterionic lipids with a well‐exposed amine headgroup, may generally reduce protein adsorption in liposomal environments, although protein‐liposome binding still significantly depends on the specific protein [32]. Controlling liposome composition not only reduces protein corona formation but can also be used to promote the selective adsorption of specific proteins, thereby enabling the coating of liposomes with targeting ligands [21]. For instance, a high abundance of DOTAP in liposomes attracts vitronectin from plasma, which has a high affinity for integrins overexpressed on various cancer cells [30]. Similarly, DOPE‐rich liposomes attract apolipoproteins that can be recognized by scavenger receptors on cancer cells [83].

Note that differences in molecular structures and system sizes between our simulations and experiments preclude direct quantitative comparison. For example, the CG model used here employs Gō models that allow structural flexibility of proteins and more accurate conformational representations but still cannot accurately predict secondary structures [84, 85, 86], which are known to be important for protein‐surface interactions [87, 88]. This CG model imposes constraints that prevent protein unfolding, which may lead to an artificial underestimation of binding and effectively exclude irreversible protein‐liposome binding pathways mediated by hydrophobic interactions associated with protein denaturation [89]. In addition, it cannot be ruled out that larger liposomes (>100 nm) might result in different effects of liposome composition and size. Different liposome sizes (i.e., curvature) might influence the kinetics of protein binding and detachment from liposomes [90], which is, while worthwhile, beyond the scope of this study, and we hope to report on elsewhere. Despite these limitations, our simulation findings not only agree well with experimental observations but also provide mechanistic insight into the influence of liposome composition and size, as well as the Vroman effect of competitive protein adsorption at the early stage of corona formation.

## 3 Conclusions

We performed CG MD simulations for the adsorption of plasma proteins (SA, IgG, C3, and FG) onto cationic, anionic, and zwitterionic liposomes. Liposomes composed of cholesterol and phospholipids such as DOPC, DOPE, DOPG, and DOTAP were simulated in water with 0.15 m NaCl for 300 ns, showing equilibrated sizes of approximately 26 and 36 nm. Starting from initial configurations containing 15 SA, 10 IgG, 10 C3, or 4 FG proteins randomly placed around a liposome, proteins adsorb to liposomes, to an extent dependent on liposome composition and size. Proteins adsorb more substantially onto charged liposomes than onto zwitterionic ones via electrostatic interactions with lipid headgroups and hydrophobic interactions with lipid tails, with adsorption following the order of cationic > anionic > zwitterionic liposomes. For zwitterionic liposomes, protein adsorption is more pronounced on liposomes with smaller headgroups. Fewer SA proteins adsorb onto smaller (26 nm) liposomes than onto larger (36 nm) liposomes because their relatively weak binding makes them more readily detached at higher bilayer curvature. These effects of liposome composition and size are consistent with experimental observations.

To reproduce the competitive adsorption of different plasma proteins in human plasma, we also simulated a mixture of 20 SA, 8 IgG, 2 C3, and 2 FG proteins adsorbed onto cationic, anionic, and zwitterionic liposomes, showing preferential adsorption of cationic and anionic proteins onto oppositely charged liposomes, which indicates the role of liposome and protein electrostatics. In particular, diffusivity and binding free‐energy calculations show that liposome‐protein interactions are energetically stabilized when abundant SA is replaced by high‐affinity IgG or C3 through electrostatic and hydrophobic interactions between proteins and those between proteins and lipids, depending on the protein type, which supports the Vroman effect regarding competitive protein adsorption at the early stage of corona formation.

## 4 Experimental Section

All simulations and analyses were performed using the GROMACS‐2024.5 simulation package [91, 92, 93, 94] with the “MARTINI 3” CG force field (FF), which represents a few (two to four) heavy atoms as a single CG bead [84, 95, 96, 97]. Coordinates for SA, IgG, C3, and FG were obtained from the Protein Data Bank (PDB codes: 1AO6 [98], 1HZH [99], 2A73 [100], and 3GHG [101], respectively) and then modeled using the Martinize2 program [85] with the GōMartini 3 approach [86]. This Gō‐like model combines a virtual‐site implementation of Gō models with Martini 3 [85, 86], enhancing the structural flexibility of proteins and thus enabling a more accurate representation of conformational changes and mechanical stability, particularly for protein interactions with membranes, ligands, and small molecules [97, 102, 103, 104, 105]. Potentials for DOPC, DOPE, DOPG, DOTAP, and cholesterol were taken directly from the MARTINI 3 lipid [97] and cholesterol [106] FFs.

A physiological temperature of 310 K and a pressure of 1 bar were maintained by applying a velocity‐rescaling thermostat [107] and a cell‐rescaling barostat [108] in the NPT ensemble. A real‐space cutoff of 1.1 nm was applied for Lennard–Jones and Coulombic interactions, with reaction‐field electrostatics and a dielectric constant of 15. Each liposome‐protein system was simulated for 2 µs with a timestep of 20 fs, resulting in a total simulation time of 88 µs for 44 distinct liposome‐protein systems, conducted on computational facilities supported by the National Supercomputing Center with supercomputing resources, including technical support (KSC‐2025‐CRE‐014).

### 4.1 Equilibration of Liposomes

26 and 36 nm‐sized liposomes, composed of cholesterol and phospholipids such as DOPC, DOPE, DOPG, or DOTAP, were generated using the TS2CG membrane‐builder tool developed by Pezeshkian et al. within the framework of the MARTINI FF [109, 110]. The phospholipid‐to‐cholesterol ratio was set to 7:3, as the inclusion of 30∼40 mol% cholesterol has been experimentally shown to improve liposome stability [73, 111]. Each 36 nm liposome, consisting of 7706 lipids and 3302 cholesterol molecules, was solvated with ∼1 640 000 CG water beads (corresponding to ∼6 560 000 real water molecules) in a cubic periodic box of size 60 nm per side (Table 1). Each 26 nm liposome contained 3287∼3564 lipids and 1408∼1526 cholesterol molecules and was solvated with ∼960 000 CG water beads (corresponding to ∼3 840 000 real water molecules) in a cubic periodic box of size 50 nm per side. To neutralize the system and mimic a physiological ionic concentration of 0.15 m Na^+^Cl^−^, sufficient counterions were added, together with additional 19 500 and 11 300 Na^+^/Cl^−^ ions for the 36 and 26 nm‐sized liposomes, respectively. Several energy‐minimization steps and short equilibration runs were performed to allow water and ions to pass through the liposome pore until it closed. All liposomes were then equilibrated for 300 ns with a timestep of 20 fs and subsequently used as starting configurations for simulations of liposome‐protein mixtures.

### 4.2 Simulations of Single Protein Species Around the Liposome

Either 15 SA, 10 IgG, 10 C3, or 4 FG proteins were randomly positioned around 26 and 36 nm‐sized liposomes composed of DOPC, DOPE, DOPG, or DOTAP, maintaining a minimum distance of 2 nm from the liposome surface or other proteins, with overlapping water and ions removed, resulting in 32 distinct liposome‐protein systems with a total simulation time of 64 µs (Table 1).

### 4.3 Simulations of Four Protein Species Around the Liposome

Considering the abundance of SA and IgG plasma proteins in human plasma [13, 79], a mixture of 20 SA, 8 IgG, 2 C3, and 2 FG proteins was randomly positioned around the 36 nm‐sized liposome composed of DOPC, DOPE, DOPG, or DOTAP, maintaining a minimum distance of 2 nm from the liposome surface or other proteins, with overlapping water and ions removed. Note that a simulation time of 2 µs is considered relatively long but may not be sufficient for full convergence of the protein corona composition. To address this issue, we performed three independent simulations (with different initial distributions of proteins around the liposome) for each liposome and observed similar qualitative trends across all replicates (Figures 11, 12, 13, 14), although the quantitative values differ to some extent. Therefore, 12 distinct liposome‐protein systems were simulated for a total simulation time of 24 µs (Table 1).

### 4.4 Diffusivity Calculations

Diffusion coefficients of individual proteins were obtained by calculating the slope of their mean‐square displacement (*D*_PBC_), corrected for finite‐size effects using the formula *D* = *D*_PBC_ + *k*_B_ *Tξ*/6π*ηL* [112], where *k*_B_ is Boltzmann's constant, *L* is the equilibrated cubic‐box length, *ξ* = 2.837297, and *η* is the solution viscosity (taken to be 0.72 cP at 310 K for MARTINI CG water) [113]. Because CG dynamics is faster than all‐atom dynamics due to smoother interactions, a rate‐scaling factor of ∼4 is often applied to account for this speed‐up [95]. However, the appropriate scaling factor may vary depending on the molecule [114, 115] and thus was not applied in this work. Therefore, the calculated diffusion coefficients should be interpreted with caution and considered only for qualitative comparison within the CG models used here.

### 4.5 Calculations of Binding Free Energies

Binding free energies between liposomes and proteins were computed using the MM‐PBSA method as implemented in GROMACS [81, 82]. In brief, the binding free energy (Δ*G*_bind_) was determined from the difference between the total free energy of the liposome‐protein complex and the sum of the free energies of the unbound liposome and protein in solvent: Δ*G*_bind_ = *G*_complex_ − (*G*_liposome_ + *G*_protein_). The total free energy of each entity was calculated according to *G*_complex, liposome, or protein_ = 〈*E*_MM_〉 − *TS* + 〈*G*_solvation_〉, where 〈*E*_MM_〉 represents the average molecular mechanics potential energy in vacuum (= *E*_vdW_ + *E*_elec_ + *E*_bond_ + *E*_angle_ + *E*_dihedral_), *TS* is the entropic contribution in vacuum, and 〈*G*_solvation_〉 is the solvation free energy, i.e., the energy required to transfer a solute from vacuum into solvent. The latter is decomposed into polar (*G*_polar_) and nonpolar (*G*_nonpolar_) contributions, corresponding to electrostatic and non‐electrostatic terms, respectively. A solute dielectric constant of 2 was employed for the calculation of *G*_polar_, as this value has previously been shown to yield binding free energies of protein complexes in good agreement with experimental data [82, 116]. The nonpolar contribution (*G*_nonpolar_) was estimated from the solvent‐accessible surface area, using a probe radius of 0.235 nm for CG simulations [96]. Free energy evaluations were performed every 100 ns for 2 µs‐long CG simulations.

## Conflicts of Interest

The author declares no conflicts of interest.

## Data Availability

The data that support the findings of this study are available on request from the corresponding author. The data are not publicly available due to privacy or ethical restrictions.;
